# Seasonality of Children’s Height and Weight and Their Contribution to Accelerated Summer Weight Gain

**DOI:** 10.3389/fphys.2022.793999

**Published:** 2022-05-10

**Authors:** Jennette P. Moreno, Salma Musaad, Hafza Dadabhoy, Tom Baranowski, Stephanie J. Crowley, Debbe Thompson, Tzuan A. Chen, Craig A. Johnston

**Affiliations:** ^1^ USDA/ARS Children’s Nutrition Research Center, Baylor College of Medicine, Pediatrics-Nutrition, Houston, TX, United States; ^2^ Biological Rhythms Research Laboratory, Department of Psychiatry and Behavioral Sciences, Rush University Medical Center, Chicago, IL, United States; ^3^ HEALTH Research Institute, University of Houston, Houston, TX, United States; ^4^ Department of Psychological, Health, and Learning Science, University of Houston, Houston, TX, United States; ^5^ Department of Health and Human Performance, University of Houston, Houston, TX, United States

**Keywords:** seasonal variation, growth, weight gain, standardized BMI, elementary school children. seasonality of Children’s height and weight

## Abstract

**Background:** While children have been shown to have increased BMI during the summer compared to the school year, it is not known if this may be due to seasonal variations in height or weight separately.

**Methods:** Trained nurses measured heights (cm) and weights (kg) in a cohort of Kindergarteners (*n* = 7648) twice per year from the beginning of kindergarten through 5th grade. Variation in height and weight by season (school year *vs*. summer) was examined using separate mixed-effects models. Season, sex, and BMI trajectory group were tested as fixed effects. Random effects included repeated measurements of time, students nested within a school, intercept, and slope for growth over time. Similar models using BMIz as the outcome examined the interaction of height or weight with season.

**Results:** The rate of height gain was greater during the school year (∼Sept to April) compared to summer (∼April to Sept) (β = -0.05, SE = 0.013, *p* < 0.0001). The rate of weight gain did not differ seasonally. Height gain was more strongly associated with increased BMIz during summer compared to the school year (β =.02, SE = 0.005, *p* <0 .0001), mainly among children who remained healthy weight throughout elementary school (β = 0.014, SE = 0.003, *p* < 0.0001) and those who transitioned to a healthier weight status (β = 0.026, SE = 0.008, *p* = 0.004). We found a similar seasonal effect for the association between weight with BMIz among children who maintained a healthy weight status (β = 0.014, SE = 0.014, p < 0.0001).

**Conclusion:** This study indicates seasonality in children’s height gain, gaining height at a faster rate during the school year compared to the summer, while weight gain remained relatively more consistent throughout the year. Seasonality in height and weight gain had the greatest impact on BMIz among children with a healthy weight status. Future research with more frequent measurements is needed to better understand the seasonal regulation of children’s growth and weight gain.

## Introduction

Numerous studies have identified that elementary school children increase their standardized BMI (BMIz) during the summer break from school at a faster rate compared to the school year (von Hippel et al., 2007; von Hippel et al., 2015; McCue et al., 2013; Moreno et al., 2015; Moreno et al., 2013; Smith et al., 2009; Kato et al., 2012; Kobayashi and Kobayashi, 2006; Isojima et al., 2018). In our 5-year longitudinal study, these gains contributed to increased rates of overweight and obesity by fifth grade. These collective findings have spurred enthusiasm for developing summertime obesity prevention programs focusing on combating the obesogenic summer environment (Tanskey et al., 2018).

Evidence of seasonal changes in the rate of children’s height and weight gain have been reported in studies as early as the 1700’s to the present (Burk, 1898; Marshall, 1937; Bogin, 1999; Yokoya et al., 2012; Baranowski et al., 2014; Dalskov et al., 2016; De Leonibus et al., 2016; Isojima et al., 2018; Moreno et al., 2020). These studies suggest that acceleration in children’s growth and weight gain may be influenced by seasonal effects such as changes in the light-dark cycle, temperature, or humidity rather than the structure of the school versus the summer environment (Brazendale et al., 2017), resulting in accelerated height gain in the spring and early summer and possible accelerated weight gain in the late summer and fall (Moreno et al., 2020). Most studies examining school-summer differences in the rate of BMI change have failed to control or accounted for the potential impact of seasonal effects on children’s linear growth and weight gain patterns (Moreno et al., 2020).

The objectives of the current study were to examine 1) seasonal variation in height and weight gain in our 5-year longitudinal study and 2) the impact of seasonal variation in height or weight on children’s change in BMIz. Findings may have important implications for whether the summer break from school is a critical time to prevent accelerated weight gain in children and offer further insight into possible factors that regulate the timing of children’s growth and weight gain.

## Materials and Methods

This is a secondary analysis of a 5-year longitudinal study that followed a cohort of kindergarteners from 41 elementary schools within a Southeast Texas independent school district (1). In the United States, children generally enter kindergarten at age 5 and are typically age 10 at the beginning of 5^th^ grade. Nurses, trained by research staff, measured children’s heights (in) and weights (lbs) twice per year from the beginning of kindergarten through 5^th^ grade (mean dates of assessment: September 17 and April 20). The methods have previously been described in detail (Moreno et al., 2013). Data were collected as administrative data which the school district allowed the researchers to use anonymously. The Fort Bend Independent School District and the Institutional Review Board of the Baylor College of Medicine approved the study.

Tanita Accustat stadiometers were installed in all schools and were also provided with a Tanita HD-351 Digital scale. Heights and weights were obtained with the children wearing light clothing and no footwear. The training of nurses has been described elsewhere (Moreno et al., 2013). Heights and weights were converted to centimeters and kilograms. Height, weight, sex, and age of the child at measurement were used to calculate BMIz and BMI percentile using the SAS program provided by the CDC (Centers for Disease Control and Prevention, 2007).

### Data Analyses

The distributions of continuous variables were examined using measures of central tendency and normality probability plots. Comparisons of categorical data were performed using the Chi-square test or Fisher’s exact test. Continuous data were compared across groups using the *t*-test or Wilcoxon 2-sample test with t-approximation. Variables used to adjust the models were selected based on empirical evidence of differences in weight status or seasonal patterns in weight gain or obesity-related behaviors across various demographic variables available in this dataset (Moreno et al., 2013; Franckle et al., 2014; Moreno et al., 2015; Chen et al., 2016; Sallis et al., 2019; Chang et al., 2021; Min et al., 2021). Group-based trajectory modeling was conducted using the PROC TRAJ macro in SAS (Jones et al., 2001). BMI trajectory group membership was estimated using data collected at up to 11 time points for height (cm) or weight (kg) and the binary outcome of overweight/obese (=1) *vs*. non-overweight/obese (=0). Evaluation of model fit to determine the number of trajectories was conducted as previously described in (Chen et al., 2016). The final set of variables used to adjust the models consisted of age (months), season, sex, race/ethnicity, and BMI trajectory group membership.

**FIGURE 1:**
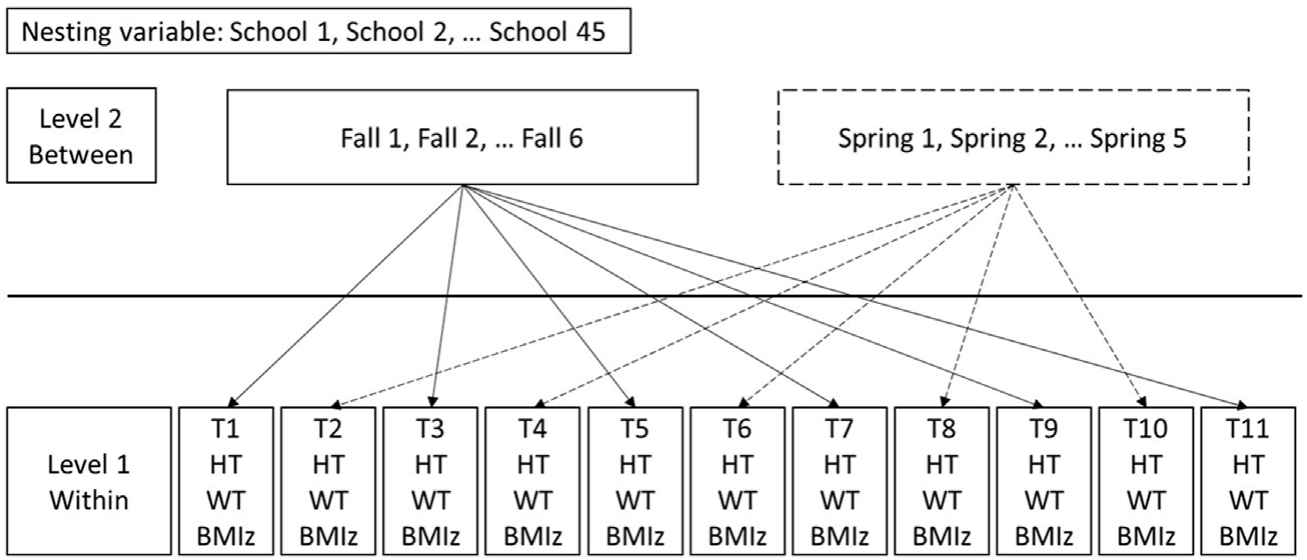
Mixed effect model structure.

Considering that person effects such as height or weight are level 1 and clustered within level 2 effects (season), centering was conducted to partition the influence of within- and between-season effects (Figure 1) (Enders and Tofighi, 2007). Additional information about centering procedures and interpretation is provided in the online Supplementary Material.

To achieve the first objective (i.e., examine evidence of seasonal variation in height and weight gain), the variation in height (cm) or weight (kg) by season (school year *vs*. summer) was examined using separate mixed-effects models (Figure 1). For each outcome (height or weight), the mixed models were tested in stages, beginning with the unconditional model (no covariates), followed by adding random intercept and covariates, followed by the slope (age (months)). Season was determined based on whether measurements were collected in the fall semester (times 1, 3, 5, 7, 9, and 11 indicating the summer effect) or spring semester (times 2, 4, 6, 8, and 10, indicating the school year effect). Age was added as a random slope to test whether average growth in the outcome varied across students. Covariates were added consisting of level 1 covariates (sex, race/ethnicity, age, age^2^), level 2 covariates (season), and the season x age interaction. Differences by season in the rate of change in the outcome of height (cm) or weight (kg) conditional on racial/ethnic and BMI trajectory groups were tested using the following 3-way interactions: 1) season (Fall *vs*. Spring) x age (months) x race/ethnicity and 2) season (Fall *vs*. Spring) x age (months) x trajectory group. See Supplementary Material for additional detail.

To achieve the second objective (i.e., examine the impact of seasonal variation in height or weight on children’s change in BMIz), BMIz was the dependent variable and height-for-age Z scores or weight-for-age Z scores rather than the raw values (as done in the first objective). This was done to be on the same scale as the outcome of BMIz. To disentangle the within seasonal (person-specific, level 1) effects from the between seasonal (level 2), two interactions with season were tested. To estimate the cross-level interaction we tested the season x season centered height or weight. To estimate the between seasonal effect we tested the season x season mean height or weight interaction. Other covariates were sex, ethnicity, age, age^2^, season, mean height (or weight) within a season, and height (or weight) centered within season. The difference in the rate of change in the outcome of BMIz due to the season x season mean height interaction was examined across race/ethnicity and trajectory groups using the following 3-way interaction 1) season (Fall *vs*. Spring) x season mean height (cm) x race/ethnicity and 2) season (Fall *vs*. Spring) x season mean height (cm) x trajectory group.

To reduce the chance of false discovery caused by multiple testing, the significance level for the study was adjusted using the conservative Bonferroni correction to control for 3 different outcomes of height, weight, and BMIz (2-tailed *p* <0 .016). Due to the complexity of the covariance structure, the denominator degrees of freedom were approximated (specified null distribution for test statistic of fixed effects) using the Satterthwaite method (Banh, Blozis, 2015). Underweight children and Native Americans were excluded due to low representation in the sample. All analyses were conducted using Statistical Analysis Software (SAS) version 9.4 (SAS Institute, Cary, NC, United States ).

## Results

The characteristics of the overall sample at study entry and sample present at baseline are described in Table 1. This cohort of kindergarteners was composed of slightly more males than females at baseline. At baseline, the sample had equal proportions of children from Black, white, and Hispanic racial/ethnic backgrounds (26–28%) while children with Asian backgrounds composed 18% of the sample at baseline. A higher proportion of children of Black or Hispanic descent matriculated into the study after baseline.

**TABLE 1:** Characteristics of the baseline sample and all sample at study entry [means or n (s.d. or %)].

	All sample at study entry	Present at baseline
N	7648	3273
Age (years)	7.24 (1.85)	5.66 (0.34)
Females	3732 (48.80)	1601 (48.92)
Race/Ethnicity		
Asian	1306 (17.08)	586 (17.90)
Black	2753 (36.00)	926 (28.29)
Hispanic	1978 (25.86)	858 (26.21)
White	1611 (21.06)	903 (27.59)
Anthropometrics		
Height for Age Z score	0.10 (1.05)	0.15 (1.03)
Weight for Age Z score	0.42 (1.10)	0.36 (1.06)
BMI Z score	0.56 (1.04)	0.52 (1.02)
BMI Percentile	64.74 (28.21)	63.51 (27.75)

Note: The overall sample includes all children who provided data. The baseline sample only includes participants present at baseline. The sample missing baseline data includes those who were either missing during the baseline data collection or matriculated into the 2005 kindergarten cohort after baseline. The sample with complete data provided height and weight measurements at all time points.

### Trajectory Group Membership Based on Probability of Having a BMI ≥85^th^ Percentile

**FIGURE 2:**
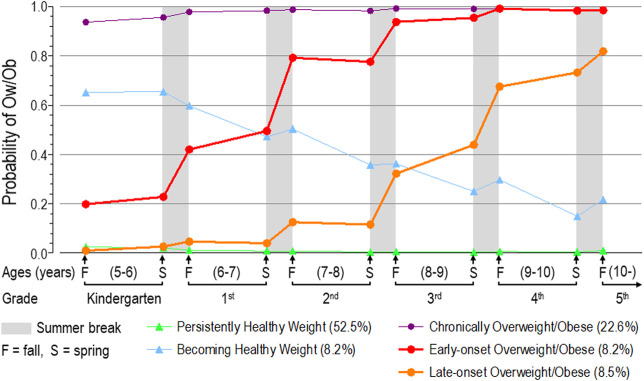
BMI trajectories of probability of overweight/obesity (BMI ≥85th percentile).

Because we had previously examined trajectory group membership only among individuals with complete data at each time point, for the current paper, we reconstructed these trajectory groups using the full data (Chen et al., 2016). Similar to our previous findings (Chen et al., 2016), a 5-trajectory group model was chosen based on the model having converged, the largest BIC, a change in BIC of at least 10 points, posterior probabilities >0.7, each group was comprised of at least 5% or more of the sample, and the groups were interpretable (Figure 2).

The 5-group solution obtained in the current analyses followed the same pattern observed previously (Chen et al., 2016). Most children (52.5%) were grouped in the Persistently Healthy Weight group while 22.6% were classified as Chronically Overweight/Obese. About 8.2% transitioned from a heavier weight status in kindergarten to a healthier weight status over the course of elementary school (Becoming Healthy Weight Group). Two groups transitioned from a healthy weight status to an unhealthy weight status, with one group beginning this transition during the summer after kindergarten (8.2%; Early-onset Overweight/Obese) and the other beginning during the summer after 2^nd^ grade (8.5%; Late-onset Overweight/Obese).

### Seasonal Variation in Gain in Height (cm)

**FIGURE 3:**
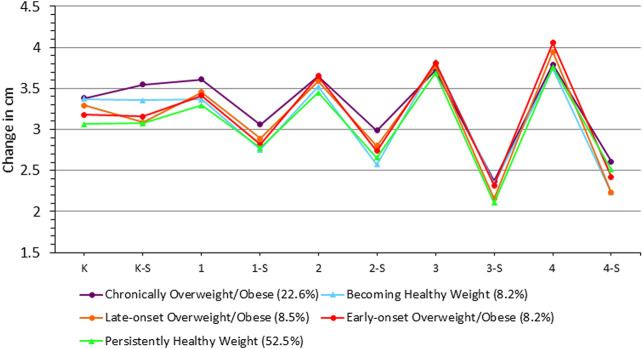
Seasonal variability in height gain across trajectory groups (means adjusted for age (months), season, sex, and race/ethnicity). K: Kindergarten School Year; K‐S: Kindergarten Summer; 1: 1st Grade School Year; 1‐S: 1st Grade Summer; 2: 2nd Grade School Year; 2‐S: 2nd Grade Summer; 3: 3rd Grade School Year; 3‐S: 3rd Grade Summer; 4: 4th Grade School Year; 4‐S: 4th Grade Summer.

When looking at the main effect of season alone, we found that children were taller in the summer than the school year by an average of 2.6 cm (β = 2.608 [95% confidence interval (CI) = 1.314, 3.902], *p* < 0.0001; Table 2). When examining the difference in height gain across seasons (seasonality), we found a significant effect of season on the increase in height, suggesting that children increased their height at a slower rate during the summer than during the school year {β = -0.055 [95% confidence interval (CI) = −0.081, −0.029], *p* < 0.0001; Figure 3}. Specifically, children’s height increased faster during the school year compared to the summer by an average rate of 0.05 cm per month. When examining differences in height gain across trajectory groups, we observed that the Chronically Overweight/Obese grew an average of 0.11 cm more per month than the Persistently Healthy Weight group (β = 0.111 ([95% confidence interval (CI) = 0.077, 0.145], *p* < 0.0001). However, there were no differences in mean height between the summer and school year across trajectory groups. However, height increased at a slower rate in the summer than school year among the Chronically Overweight/Obese {β = −0.05 [95% confidence interval (CI) = −0.082, −0.021], *p* < 0.0001} and the Persistently Healthy Weight (β = -0.041 ([95% confidence interval (CI) = -0.062, −0.019], *p* < 0.0001). No seasonality in height increases was observed among the late-onset, early-onset, and becoming healthy weight trajectory groups. When the same model was evaluated with the outcome of height-for-age Z scores rather than raw height (cm), we obtained comparable results.

**TABLE 2:** Mixed model findings for effect of season on the outcome of height (cm).

	β	SE	95% CI	P
Child sex (Female *vs*. Male)	−1.208	0.119	−1.441, −0.975	<0.0001
Slope (age in months)	0.571	0.016	0.540, 0.602	<0.0001
Season (Fall *vs*. Spring)	2.608	0.660	1.314, 3.902	<0.0001
Age x season (Fall *vs*. Spring)	−0.055	0.013	−0.081, −0.029	<0.0001
Age (months) x trajectory group				
Late-onset Overweight/Obese	0.011	0.027	−0.043, 0.064	0.694
Early-onset Overweight/Obese	0.039	0.029	−0.019, 0.096	0.189
Chronically Overweight/Obese	0.111	0.017	0.077, 0.145	<0.0001
Becoming Healthy Weight	0.032	0.027	−0.021, 0.086	0.232
Persistently Healthy Weight (reference)	−	−	−	−
Trajectory group x season (Fall *vs*. Spring)				
Late-onset Overweight/Obese	−0.146	1.155	−2.409, 2.118	0.899
Early-onset Overweight/Obese	−0.079	1.254	−2.537, 2.378	0.949
Chronically Overweight/Obese	0.494	0.708	−0.894, 1.882	0.486
Becoming Healthy Weight	−1.782	1.125	−3.986, 0.423	0.113
Persistently Healthy Weight (reference)	**−**	**−**	**−**	**−**
Age (months) x season (Fall *vs*. Spring)				
Late-onset Overweight/Obese	−0.038	0.022	−0.094, 0.018	0.407
Early-onset Overweight/Obese	−0.037	0.024	−0.099, 0.026	0.672
Chronically Overweight/Obese	−0.052	0.012	−0.082, −0.021	<0.0001
Becoming Healthy Weight	−0.003	0.021	−0.058, 0.052	1.000
Persistently Healthy Weight	−0.041	0.008	−0.062, −0.019	<0.0001

SE, standard error; CI, confidence interval.

Fall coincides with summer and spring coincides with school year. Significance was determined using a 2-tailed *p* <0 .016 to reduce the chance of false discovery. The model was adjusted for race/ethnicity and school was used as a nesting factor. The percent of group membership (out of 7743 individuals) for the Late-onset Overweight/Obese = 8.5%, Early-onset Overweight/Obese = 8.2%, Chronically Overweight/Obese = 22.6%, Becoming Healthy Weight = 8.2%, and Persistently Healthy Weight = 52.5%.

### Association of Height-For-Age Z Scores With the Outcome of BMIz

Table 3 presents the mixed model results for testing the association of height-for-age Z scores with the outcome of BMIz. The association of within-child height-for-age Z scores with change in BMIz did not vary by season (i.e., the influence of children’s height gain on BMIz was not moderated by season). This was evidenced by a non-significant cross-level interaction of season (level 2) x height-for-age Z scores centered within season (level 1) (β = 0.011 [95% CI = -0.007, 0.029], *p* = 0.238). In contrast, the association of season mean height-for-age Z score with change in BMIz varied between seasons, suggesting that mean height-for-age Z scores within a season (level 2) was positively associated with change in BMIz and this effect was slightly stronger in the summer than the school year (β = 0.021 [95% CI = 0.012, 0.030], *p* < 0.0001).

**TABLE 3:** Mixed model findings testing the association of height-for-age Z scores with the outcome of BMIz.

	β	SE	95% CI	P
Season (Fall *vs*. Spring)	0.003	0.005	−0.006, 0.011	0.576
Within-child height-for-age Z score	−0.313	0.008	−0.329, −0.297	<0.0001
Within-child height-for-age Z score x season (Fall *vs*. Spring)	0.011	0.009	−0.007, 0.029	0.238
Slope (age in months)	−0.003	0.001	−0.006, −0.002	<0.001
Child sex (Female *vs*. Male)	−0.052	0.012	−0.075, −0.029	<0.0001
Mean height-for-age Z score within season	−0.004	0.013	−0.029, 0.021	0.764
Mean height-for-age Z score within season x season (Fall *vs*. Spring)	0.021	0.005	0.012, 0.030	<0.0001
Trajectory group				
Late-onset Overweight/Obese	0.862	0.025	0.813, 0.912	<0.0001
Early-onset Overweight/Obese	1.296	0.028	1.241, 1.349	<0.0001
Chronically Overweight/Obese	1.869	0.0156	1.839, 1.901	<0.0001
Becoming Healthy Weight	1.009	0.025	0.961, 1.058	<0.0001
Persistently Healthy Weight (reference)				
Mean height-for-age Z score within season x season (Fall *vs*. Spring)				
Late-onset Overweight/Obese	−0.003	0.008	−0.025, 0.018	1.000
Early-onset Overweight/Obese	0.004	0.009	−0.019.0.026	1.000
Chronically Overweight/Obese	−0.000	0.004	−0.011, 0.011	1.000
Becoming Healthy Weight	0.026	0.008	0.006, 0.046	0.004
Persistently Healthy Weight	0.014	0.003	0.006, 0.021	<0.0001

SE, standard error; CI, confidence interval.

Fall coincides with summer and spring coincides with school year. The within-child height-for-age Z score x season indicates the cross-level interaction between child height (level 1) and season (level 2), testing whether the association between child height and BMIz is moderated by season. The interaction of mean height-for-age Z score within season x season tests season to season effects (level 2). The percent of group membership (out of 7743 individuals) for the Late-onset Overweight/Obese = 8.5%, Early-onset Overweight/Obese = 8.2%, Chronically Overweight/Obese = 22.6%, Becoming Healthy Weight = 8.2%, and Persistently Healthy Weight = 52.5%.

To assess differences in how the association of season mean height-for-age Z score varied between seasons across trajectory groups, the 3-way interaction of mean height (cm) within a season x season (summer *vs*. school year) x trajectory group was added. In general, the differences in height between seasons were small among the trajectory groups. The difference in mean height (cm) within a season by summer *vs*. school year was highest among Becoming Healthy Weight (β = 0.026 [95% CI = 0.006, 0.046], *p* = 0.004) and Persistently Healthy Weight groups β = 0.014 [95% CI = 0.006, 0.021], *p* < 0.001). This suggests that the seasonality of height contributed to increases in BMIz among only the Persistently Healthy and Becoming Healthy Weight groups.

### Seasonal Variation in Weight Gain (kg)

**FIGURE 4:**
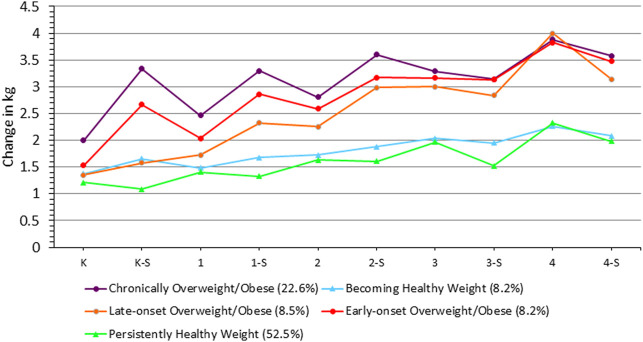
Seasonal variability in weight gain across trajectory groups (means adjusted for age (months), season, sex, and race/ethnicity). K: Kindergarten School Year; K‐S: Kindergarten Summer; 1: 1st Grade School Year; 1‐S: 1st Grade Summer; 2: 2nd Grade School Year; 2‐S: 2nd Grade Summer; 3: 3rd Grade School Year; 3‐S: 3rd Grade Summer; 4: 4th Grade School Year; 4-S: 4th Grade Summer.

As for weight (kg), the main effect of season was not significant (β = −0.246 [95% CI = -1.526, 1.034], *p* = 0.706, Figure 4). The Chronically Overweight/Obese gained on average 0.24 kg more per month than the Persistently Healthy Weight group (β = 0.238 [95% confidence interval (CI) = 0.201, 0.275], *p* < 0.0001). Similarly, the Becoming Healthy Weight gained on average 0.10 kg more per month than the Persistently Healthy Weight group (β = 0.091 [95% confidence interval (CI) = 0.033, 0.150], *p* = 0.002). In contrast, the Late-onset Overweight/Obese group gained weight at a slower rate than the Persistently Healthy Weight group by an average of -0.33 kg per month. (β = −0.329 [95% confidence interval (CI) = −0.388, −0.271], *p* < 0.0001).

When looking at the three-way interaction of change in weight by season across trajectory groups, the late-onset overweight and obese increased their weight by 0.07 kg more per month in the summer compared to the school year {β = 0.074 [95% confidence interval (CI) = 0.018, 0.129], *p* = 0.003}. When the same model was tested with the outcome of weight-for-age Z scores rather than raw weight (kg), we obtained comparable results.

### Association of Weight-For-Age Z Scores With the Outcome of BMIz

Table 4 summarizes the mixed model findings evaluating the association of weight-for-age Z scores with BMIz. The association of within-child weight-for-age Z scores with change in BMIz did not vary by season (i.e., the influence of child height gain on BMIz was not moderated by season) [β = 0.007 (95% CI = −0.004, 0.017), *p* = 0.218]. This finding indicates that the association of individual variation in weight-for-age Z scores with BMIz does not vary by season.

**TABLE 4:** Mixed model findings testing the association of weight-for-age Z scores with the outcome of BMIz.

	β	SE	95% CI	P
Season (Fall *vs*. Spring)	−0.000	0.003	−0.006, 0.006	0.989
Within-child weight-for-age Z score	1.088	0.005	1.078, 1.098	<0.0001
Within-child weight-for-age Z score x season (Fall *vs*. Spring)	0.007	0.005	−0.004, 0.017	0.218
Slope (age in months)	−0.006	0.001	−0.008, −0.005	<0.0001
Child sex (Female *vs*. Male)	−0.014	0.009	−0.032, 0.004	0.118
Mean weight-for-age Z score within season	0.794	0.012	0.774, 0.815	<0.0001
Mean weight-for-age Z score within season x season (Fall *vs*. Spring)	0.016	0.003	0.009, 0.022	<0.0001
Trajectory group				
Late-onset Overweight/Obese	0.186	0.024	0.139, 0.233	<0.0001
Early-onset Overweight/Obese	0.189	0.033	0.125, 0.254	<0.0001
Chronically Overweight/Obese	0.569	0.022	0.526, 0.613	<0.0001
Becoming Healthy Weight	0.231	0.024	0.184, 0.277	<0.0001
Persistently Healthy Weight (reference)				
Mean weight-for-age Z score within season x season (Fall *vs*. Spring)				
Late-onset Overweight/Obese	−0.003	0.009	−0.029, 0.022	1.000
Early-onset Overweight/Obese	0.001	0.011	−0.027, 0.029	1.000
Chronically Overweight/Obese	0.006	0.004	−0.005, 0.016	0.857
Becoming Healthy Weight	0.021	0.009	−0.002, 0.045	0.104
Persistently Healthy Weight	0.014	0.003	0.007, 0.020	<0.0001

SE, standard error; CI, confidence interval.

Fall coincides with summer and spring coincides with school year. The within-child weight-for-age Z score x season indicates the cross-level interaction between child weight (level 1) and season (level 2), testing whether the association between child weight and BMIz is moderated by season. The interaction of mean weight-for-age Z score within season x season tests season to season effects (level 2).

To assess differences in this significant interaction, the 3-way interaction of mean weight-for-age Z scores within a season x season (Fall *vs*. Spring) x trajectory group was added. The difference in mean weight-for-age Z scores within a season by summer *vs*. school year varied by trajectory group. Specifically, the Persistently Healthy Weight group had a greater BMIz in summer than school year β = 0.014 [95% CI = 0.007, 0.020], *p* < 0.0001). No significant school-summer differences were observed across the other trajectory groups.

To facilitate comparisons with previous findings (Moreno et al., 2013), the cumulative effects of these influences across the years of elementary school are illustrated in Supplementary Materials.

## Discussion

The influence of seasonality of children’s height and weight gain on previous findings regarding accelerated summer weight gain is unknown (von Hippel et al., 2007; von Hippel et al., 2015; McCue et al., 2013; Moreno et al., 2015; Moreno et al., 2013; Smith et al., 2009; Kato et al., 2012; Kobayashi and Kobayashi, 2006; Isojima et al., 2018). We conducted a reanalysis of a dataset in which seasonal variation affected children’s change in BMI during the school year and summer (Moreno et al., 2013; Moreno et al., 2015). We observed that children’s height and weight gain during summer contributed more strongly to BMIz than during the school year. When examining these differences across child weight trajectory groups, we found that healthy-weight children appear to be more responsive to this seasonality in both height gain and weight gain. These results suggest the impact of seasonality in height and weight involving increases in BMIz is supportive of a healthy weight status.

Overall, there was greater evidence of seasonality in children’s height than weight gain. Children gained height at a slightly faster rate during the school year compared to the summer. This pattern was most evident among the chronically overweight and obese and the Persistently Healthy Weight. These findings are consistent with a recent review indicating an association between the timing of increases in children’s height gain and changes in the seasonal light/dark cycle while the evidence regarding weight gain was less conclusive (Moreno et al., 2020). The exact seasonal timing of when children experience the greatest increases in height cannot be determined in the current study which only included two measurements per year (i.e., at the beginning and end of the school year). The observed seasonality in height gain may explain improvements in children’s BMIz observed during the school year and larger increases in BMIz during summer. Specifically, children’s height gain during summer contributed more strongly to BMIz than during the school year. This seasonal effect of height on BMI was most apparent in the Becoming Healthy Weight and Persistently Healthy Weight groups. Overall, seasonality in height appears to be associated with developing/maintaining a healthy weight status. The rate of height gain being greater during the school year (from the fall to the spring measurement), could be driven by a particular subgroup (e.g., birth month) (Weber et al., 1998; Hemati et al., 2020), behavioral differences (e.g., sleep, eating, physical activity) (Moreno et al., 2021), and light exposure patterns (Moreno et al., 2021), environmental factors (e.g., social demands), in equalities in living conditions (Wake et al., 2022), or some synergy among these factors (Moreno et al., 2019; Moreno et al., 2020).

In contrast, children’s weights did not differ across seasons and the rate of weight gain did not differ across the school year and the summer, suggesting that children gained weight at a similar rate across seasons. A lack of seasonality in children’s weight gain is consistent with studies conducted in Guatemala that similarly observed evidence of seasonality in children’s height (Bogin, 1978), but not weight (Bogin, 1979). When examining these patterns across trajectory groups we found evidence of a seasonal effect on weight gain among the Late-onset Overweight/Obese group. This may indicate weight increases in a more consistent manner across most children, though children in the late-onset overweight/obese group, who begin a transition to an unhealthy weight status beginning the summer after second grade, exhibit greater seasonality in their weight gain patterns. One limitation of the current study is that it included only two-yearly measurements of height and weight. A previous examination of circannual variation in children’s monthly BMIz suggested that monthly assessment of BMIz may yield different conclusions, regarding the timing of seasonal increases in BMIz than biannual measurements (Bhutani et al., 2018). Nevertheless, the lack of seasonal differences in weight are consistent with others who have failed to find evidence of seasonality in children’s weight gain (Bogin, 1979; Bogin, 1999). When examining the effect of seasonality in weight gain on BMIz, we observed an overall seasonal effect of weight on BMIz, though this effect was greatest in the Persistently Healthy Weight group. Overall, these findings suggest that increases in weight during summer are associated with the maintenance of a healthy weight status.

The strengths of this study include 5 years of repeated measurements from a large diverse cohort of elementary school children. However, the generalizability of our findings may be limited by the fact that the study took place in Houston, Texas. Houston has a temperate climate which may facilitate engagement in outdoor physical activity throughout the school year. However, summers can be quite hot and humid and may discourage children from spending time outdoors. In a previous study comparing the activity and sleep patterns of 5-8-year-olds in Houston, we found that during summer, children increased their time spent in sedentary activities and decreased their engagement in light physical activity during summer compared to the school year. It is possible that the hot summers contributed to these differences, although these differences were not related to changes in BMI during the school year and summer (Moreno et al., 2021). In addition, we found that children’s sleep midpoints shifted 1.5 h later in summer compared to the school year and having a later sleep midpoint was associated with greater increases in BMI during summer. While a change in sleep timing could affect exposure to the light/dark cycle, we found no differences in the amount of outdoor light exposure during the school year and summer, though increased exposure to outdoor light during the school year was associated with smaller changes in BMI (Moreno et al., 2021). It is unclear how the Houston climate and seasonal differences in day length may affect our current findings and whether these findings would be generalizable to different climates. However, it should be noted that our findings regarding seasonal changes in BMIz are consistent with results obtained in nationally representative samples (von Hippel et al., 2007; von Hippel and Workman, 2016). Examination of seasonal variation in children’s height and weight in datasets including monthly measurements from diverse climates and latitudes is needed to disentangle the effect of season on children’s growth. There is also a need to determine the impact of climate on children’s weight-related behaviors. These studies also suggest that season of measurement should be controlled for in studies examining change in BMI, whether it be interventional studies or epidemiological studies.

## Conclusion

This study indicates seasonality in children’s height gain, gaining height at a faster rate during the school year compared to the summer, while weight gain remained more consistent throughout the year, except among the Late-onset Overweight/Obese group. Despite the observed seasonal pattern in height gain involving greater increases during the school year than summer and an overall lack of seasonality in weight gain, children’s height and weight gain contributed more strongly to BMIz during summer than during the school year. The seasonal effect of height and weight on BMIz was evident among children who maintained healthy weight status across elementary school. Overall this suggests that the differential seasonal impact of increases in height and weight on BMIz appeared to be supportive of a healthy weight status. While previous findings regarding the contribution of accelerated summer weight gain to increased rates of overweight and obesity among elementary school students suggest the importance of prevention of accelerated summer weight gain, our findings suggest children who are at greatest risk of developing an unhealthy weight status would likely benefit from obesity prevention interventions during both the school year and summer. Future research with more frequent measurements is needed to better understand the seasonal regulation of children’s growth and weight gain.

## Data Availability

The original contributions presented in the study are included in the article/Supplementary Material, further inquiries can be directed to the corresponding author.
