# Supplementary material for: Seasonality of Children’s Height and Weight and Their Contribution to Accelerated Summer Weight Gain
Source: Front Physiol. 2022 May 10;13:793999. doi: 10.3389/fphys.2022.793999 (PMC9159375; doi:10.3389/fphys.2022.793999)
Supplement: Supplementary file 1 [file DataSheet1.pdf]

Supplementary Material

1 Supplementary Information

1.1 Centering procedures and their interpretation:

Centered within cluster (season) height and weight were calculated by taking the average for height (or weight) for each child within season and subtracting it from each individual observation for height (or weight). This method removes the influence of season (i.e., removes between-season effects on height (or weight)), producing a pure measure of within-child height or weight. Hence the interaction of a level 1 variable (i.e., height or weight) centered within season with season represents the cross-level interaction measuring the moderating effect of season on the relationship between level 1 height (or weight) on the outcome (BMIz).

Season mean height (or weight) was calculated by summing the height or weight within a season (Fall or Spring) then dividing the sum by the number of instances of height or weight in that season. This was considered a pure level 2 effect. Hence the interaction of the season mean with season independently represents the between-season (level 2) effect.

1.2 Additional information regarding the modeling of objective 1 and 2:

All lower 2-way interactions were included to satisfy the interaction hierarchy rule where all lower order interactions need to be included when testing interactions of 3-ways or higher. The parameter estimates for moderation of the season x age interaction by race/ethnicity and the season x age interaction by trajectory group were calculated using post-estimation techniques and adjusted for multiplicity using Bonferroni method. To account for the repeated subject observations (students were measured at multiple times at each season), a random effect for student with an autoregressive (first-order) correlation structure and nesting of student within schools were specified.

1.3 Explanation of the interaction terms:

For example, for the model that tested the effect of seasonal variation in height on BMIz, one interaction was season x season centered height. This interaction enabled us to determine how the association of a student's height with BMIz varied by season because it removed the source of between seasonal variation. The other interaction was season x season mean height, enabled us to explore how the association of season mean height with BMIz varied between seasons (Fall vs. Spring). Both interactions were included in the same model.

2 Supplementary Figures

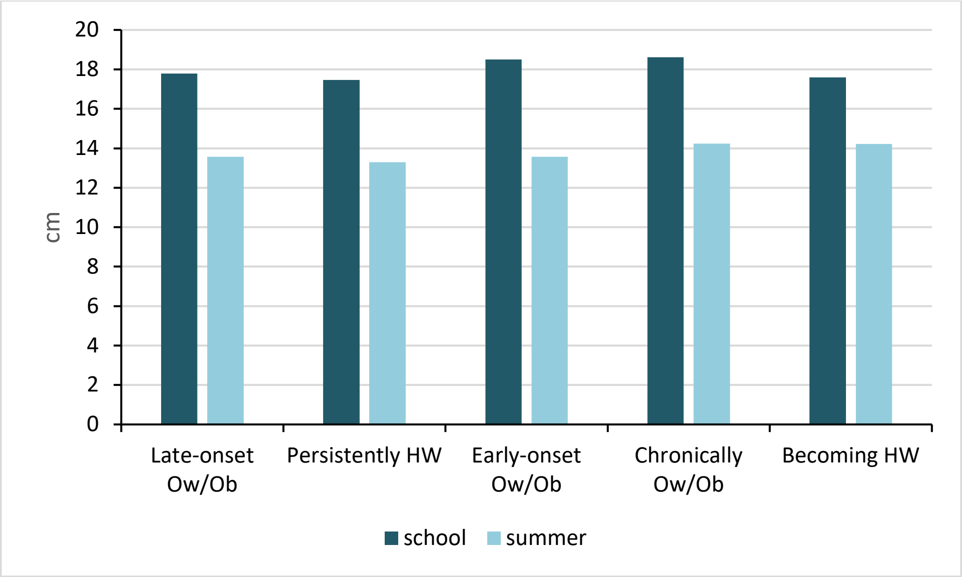

Supplementary Figure 1. Cumulative Change in Height during the School Year and Summer Across BMI Trajectory Groups. Graphed using means adjusted for age (months), season, sex, and race/ethnicity.

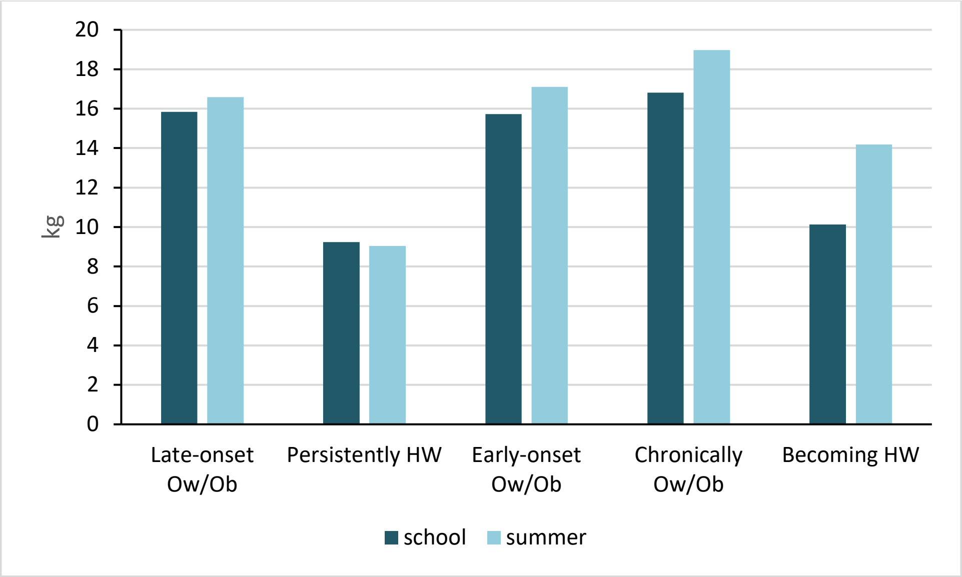

Supplementary Figure 2. Cumulative Change in Weight during the School Year and Summer Across BMI Trajectory Groups. Graphed using means adjusted for age (months), season, sex, and race/ethnicity.

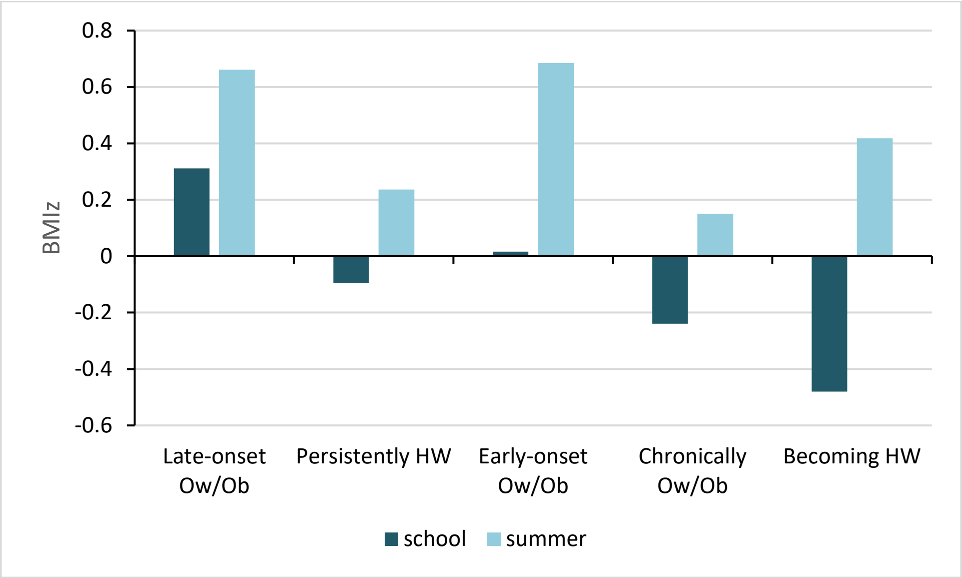

Supplementary Figure 2. Cumulative Change in BMIz during the School Year and Summer Across BMI Trajectory Groups. Graphed using means adjusted for age (months), season, sex, and race/ethnicity.
